# Targeting the cardio–neuro axis through nutrition: inflammatory mechanisms linking cardiovascular and neurodegenerative diseases

**DOI:** 10.3389/fnut.2026.1873386

**Published:** 2026-07-02

**Authors:** Darragh Curran, Neluwa-Liyanage Indika, Raymond Thomas, Niloofar Shekoohi, Andreas M. Grabrucker, Ioannis Zabetakis, Ramak Esfandi, Ronan Lordan

**Affiliations:** 1 Department of Chemical Sciences, University of Limerick, Limerick, Ireland; 2 Bernal Institute, University of Limerick, Limerick, Ireland; 3 Faculty of Medical Sciences, Department of Biochemistry, University of Sri Jayewardenepura, Nugegoda, Sri Lanka; 4 Department of Biology, Western University, London, ON, Canada; 5 College of Agriculture, Science and Education, Portland, Jamaica; 6 Department of Biological Sciences, University of Limerick, Limerick, Ireland; 7 Health Research Institute, University of Limerick, Limerick, Ireland; 8 School of Pharmacy and Biomolecular Sciences, RCSI University of Medicine and Health Sciences, Dublin, Ireland; 9 FutureNeuro Research Ireland Centre for Translational Brain Science, RCSI University of Medicine and Health Sciences, Dublin, Ireland; 10 Tissue Engineering Research Group (TERG), RCSI University of Medicine and Health Sciences, Dublin, Ireland

**Keywords:** cardiovascular disease, functional foods, neurodegeneration, nootropic foods, nutraceuticals, supplements, systemic inflammation

## Abstract

Non-communicable diseases (NCD), particularly cardiovascular diseases (CVD) and neurodegenerative diseases (ND), remain leading causes of global morbidity and mortality. Although traditionally studied in isolation, accumulating evidence indicates that these conditions are mechanistically interconnected through shared pathways, including chronic systemic inflammation, endothelial dysfunction, and dysregulated lipid metabolism. Here, we propose a cardio–neuro axis in which vascular and neurodegenerative processes are linked along a continuum that is modifiable through diet. In this perspective, we synthesise evidence linking inflammatory and neurovascular dysfunction across CVD and ND and argue that nutrition represents a primary, yet under-integrated, lever for targeting these shared mechanisms. We focus on dietary patterns and bioactive components that influence inflammation resolution, endothelial function, and metabolic homeostasis. However, despite strong mechanistic rationale, nutritional strategies for ND remain fragmented, with an overreliance on single-nutrient interventions and limited incorporation of vascular endpoints or mechanistic biomarkers. We contend that progress in this field requires a shift from reductionist approaches toward whole-diet interventions evaluated using integrated cardio-neuro outcomes, alongside stratified and personalised designs. Embedding nutrition within a unified cardio-neuro framework earlier in life may offer a scalable and mechanistically grounded strategy to reduce the burden of NCD across the life course.

## Introduction

Non-communicable diseases (NCD) are responsible for the majority of global mortality, accounting for 43 million deaths and 75% of non-pandemic-related deaths globally in 2021 (1). Among the most devastating are neurodegenerative diseases (ND) and cardiovascular diseases (CVD). While CVD remains the leading cause of death, ND, particularly Alzheimer’s disease, Parkinson’s disease, and vascular dementia are characterised by prolonged disease trajectories and increasing prevalence driven by population ageing. Despite their clinical distinction, these conditions frequently co-occur and share key risk factors, including ageing, metabolic dysfunction, and lifestyle exposures (2–4). We define the cardio-neuro axis as the bidirectional biological continuum linking cardiovascular and neurodegenerative health through shared inflammatory, vascular, metabolic, and lipid-mediated pathways. This framework provides a mechanistic basis for understanding how cardiovascular dysfunction may contribute to neurodegeneration and vice versa. While previous heart-brain axis frameworks have largely focused on physiological, haemodynamic, and neurohumoral interactions (3), the cardio-neuro axis proposed here emphasizes chronic inflammatory and metabolic disease processes and positions nutrition as a unifying and modifiable intervention strategy across both cardiovascular and ND trajectories. Currently, 55 million people worldwide live with dementia; this number is expected to reach 139 million by 2050 (5). The latest data show that dementia prevalence doubles every 5 years after the age of 60. Alzheimer’s disease accounts for approximately 70% of all dementia cases, making it an urgent and growing global challenge (6). Furthermore, more than 10 million people worldwide are estimated to live with Parkinson’s disease (7).

Despite substantial advances in cardiovascular therapeutics, including widespread statin use (8), CVD remains the leading cause of mortality worldwide and a major contributor to morbidity, placing a significant burden on global healthcare systems (8–10). Approximately 19.8 million people died from CVD in 2022, accounting for 32% of all global deaths. Of these deaths, 85% were due to myocardial infarction and stroke (9).

While primordial prevention through dietary and lifestyle modification is an effective strategy to reduce CVD risk (11, 12), emerging evidence suggests nutrition also plays a critical role in the onset and prevention of other NCD, including ND (13–15). Several inflammation-related mechanisms are shared across NCD, including ND and CVD, and may be targeted by similar therapeutic approaches (16–18).

Within this strategy, functional foods, dietary supplements, nutraceuticals, and nootropic ingredients are also under consideration for preventing and/or treating CVD and ND. Although often discussed interchangeably, these products vary in their regulatory classifications with distinct definitions (19). Functional foods are generally defined as foods that provide health benefits beyond their fundamental nutritive value. However, they do not independently treat or prevent disease, nor are they essential components of a balanced diet (19). Dietary supplements consist of nutrients or compounds designed to augment nutrient intake, avert deficiencies, and potentially offer therapeutic effects despite not being their primary purpose (20). Nutraceuticals consist of nutrients or bioactive extracts derived from food or natural sources for prophylactic or therapeutic purposes (20, 21). Nootropic foods or ingredients are biochemical compounds in foods and beverages that may improve cognition, learning, memory, and brain cellular function across the lifespan (22). Importantly, regulation and health claims related to functional foods and nutraceuticals are different around the world. In Europe, the European Food Safety Authority (EFSA) requires scientific substantiation for nutrition and health claims before they can be authorized, reflecting a generally more precautionary, pre-market approach (19, 21, 23). By contrast, in the United States, the Food and Drug Administration (FDA) regulates these products under a different framework, with more emphasis on product category, labelling, and post-market oversight rather than the same pre-market claim authorization model used in the European Union. As a result, a claim that is permitted in one market may need to be reformulated, softened, or removed in the other (19, 21, 23–25). Owing to differences in global regulatory oversight, these health claims, often closer to marketing claims, may vary considerably in scientific substantiation (26, 27).

In this article, we discuss the common aetiologies underlying CVD and ND along the cardio-neuro axis, such as shared inflammatory pathways, oxidative stress, and endothelial or neurovascular dysfunction, and provide our perspectives on how dietary and nutrient-based approaches, including functional foods, supplements, and nutraceuticals, are being explored to target these overlapping mechanisms for prevention and adjunctive therapy.

## Systemic inflammation and oxidative stress as a common underlying mechanism in cardiovascular disease and neurodegeneration

Chronic low-grade systemic inflammation is increasingly recognised as a central, shared mechanism underpinning both CVD and ND. Unlike acute inflammation, which is protective and self-limiting, persistent activation of the innate immune system contributes to a sustained inflammatory state and progressive tissue dysfunction across multiple organ systems (28). This state is characterised by sustained elevations of pro-inflammatory mediators, including cytokines such as interleukin-1β (IL-1β), interleukin-6 (IL-6), and tumour necrosis factor-*α* (TNF-α), along with acute-phase proteins including C-reactive protein (CRP). Elevated levels of these mediators are associated with increased risk of cardiovascular events and cognitive decline (17, 29, 30).

In the context of CVD, it is now understood that inflammation plays a causal role in all stages of atherosclerosis, from endothelial dysfunction and lipid deposition to plaque progression and rupture (31, 32). Endothelial activation promotes the expression of adhesion molecules, facilitating monocyte recruitment to lesion-prone intimal sites, where they differentiate into macrophages. Theses cells internalise oxidized lipoproteins to form foam cells and sustain a pro-inflammatory vascular milieu (33). Clinical trials targeting inflammatory pathways, such as IL-1β inhibition, have demonstrated reductions in recurrent cardiovascular events independent of lipid lowering, reinforcing the causal contribution of inflammation to atherothrombosis (34–36).

In many ND such as Alzheimer’s disease and Parkinson’s disease, similar inflammatory processes occur, collectively referred to as neuroinflammation, which is a defining pathological feature. Peripheral inflammatory signals can access the central nervous system (CNS) via humoral and neural pathways or through disruption of the blood–brain barrier (BBB), leading to activation of microglia and astrocytes (37). While acute glial activation may be protective, chronic activation promotes the release of neurotoxic cytokines, reactive oxygen species (ROS), and complement factors, contributing to synaptic dysfunction, and neuronal loss (38–40). In Alzheimer’s disease, chronic neuroinflammation contributes to amyloid-*β* accumulation and tau pathology (41, 42), whereas in Parkinson’s disease inflammatory processes interact with *α*-synuclein aggregation and dopaminergic neuronal degeneration (43, 44). In vascular dementia, endothelial dysfunction, cerebral small vessel disease, and chronic cerebral hypoperfusion represent particularly important mechanisms linking cardiovascular and neurodegenerative pathology.

Systemic inflammation is closely linked to vascular dysfunction in both peripheral and cerebral circulation. Chronic endothelial activation reduces nitric oxide bioavailability, increases oxidative stress, and contributes to chronic cerebral hypoperfusion and impaired neurovascular coupling, while also disrupting BBB integrity and enabling circulating cytokines and immune cells to enter the central nervous system to amplify neuroinflammatory cascades (45). This interplay between vascular dysfunction, impaired neurovascular coupling, cerebral hypoperfusion, BBB disruption, impaired neurovascular coupling, and inflammation represents a key mechanistic bridge linking cardiovascular and ND processes (46, 47).

Lipid biology further integrates these processes across the cardio-neuro axis. Dysregulated lipoproteins and altered membrane lipid composition influence membrane fluidity, lipid raft organisation, and inflammatory signalling (48). Diets high in saturated fats promote pro-inflammatory pathways, including NF-κB activation, while impairing resolution. In the CNS, disrupted lipid homeostasis compromises synaptic integrity and myelination, contributing to impaired neuronal function and pathological protein aggregation (49).

**Figure 1:**
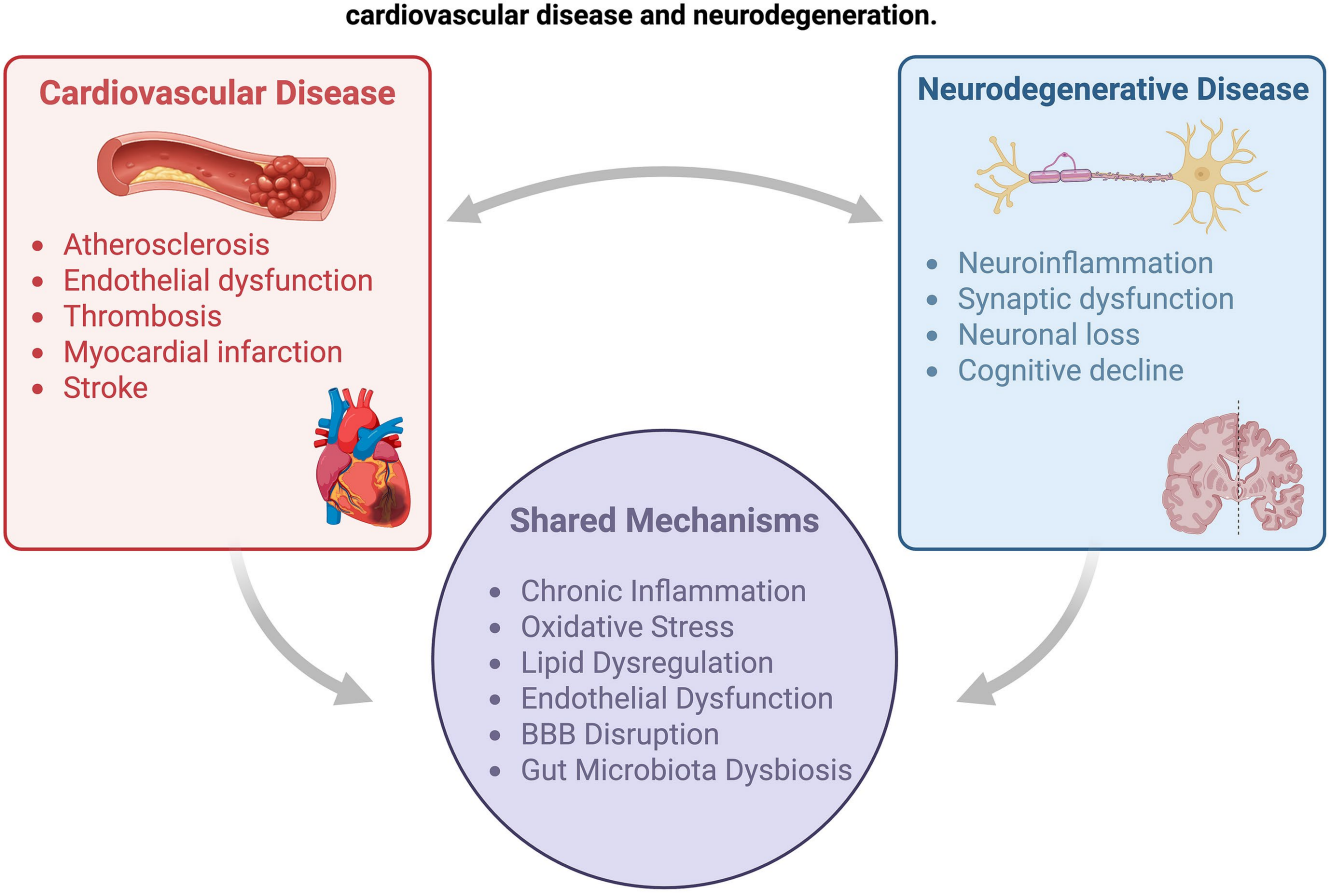
Conceptual overview showing the shared mechanisms linking cardiovascular diseases (CVD) and neurodegenerative diseases (ND).

These processes are interconnected within a neuro-cardiac continuum, where vascular inflammation impairs cerebral perfusion and BBB function, and neuroinflammation further exacerbates systemic metabolic and vascular dysfunction (50). Shared factors such as obesity, insulin resistance, dyslipidaemia, poor diet, and gut microbiota dysbiosis reinforce this overlap between cardiovascular and ND (51, 52). Collectively, systemic inflammation is a unifying, targetable driver of both CVD and ND, linking vascular dysfunction, BBB disruption, and lipid-mediated inflammatory signalling (Figure 1), providing a strong rationale for dietary and nutrient-based interventions.

Oxidative stress is a key contributor to both CVD and ND, linking vascular dysfunction with neuronal damage through shared pathological mechanisms (53, 54). Reduced activity of paraoxonase 1 (PON1), an HDL-associated antioxidant enzyme that prevents lipid peroxide accumulation in LDL, has been reported in Alzheimer’s disease, Parkinson’s disease, and coronary heart disease (55–57). Similarly, lipid peroxidation markers such as malondialdehyde (MDA) and 4-hydroxynonenal (4-HNE) are implicated in coronary heart disease and ND (58–61). Mitochondrial dysfunction and endoplasmic reticulum (ER) stress are closely linked to chronic inflammation and oxidative stress in both CVD and ND. Impaired mitochondrial bioenergetics promotes ROS production and inflammatory signalling, whereas unresolved ER stress activates pathways such as the unfolded protein response that contribute to vascular dysfunction, neuroinflammation, and cell death (62–64).

In coronary heart disease, excess reactive oxygen species (ROS) promote oxidation of LDL to oxLDL, driving foam cell formation, endothelial activation, inflammation, and plaque instability (65, 66). Comparable oxidative mechanisms occur in ND, where mitochondrial dysfunction, lipid peroxidation, neuroinflammation, and impaired antioxidant defences contribute to neuronal injury (67). In vascular cognitive impairment and dementia, oxLDL disrupts endothelial function and sustains neuroinflammation, while in Alzheimer’s disease oxidative stress interacts with amyloid-*β* and tau pathology (68, 69). In Parkinson’s disease, dopaminergic neurons are particularly susceptible due to dopamine oxidation, iron accumulation, glutathione depletion, and mitochondrial dysfunction, all of which increase ROS production and neuronal death (70). Collectively, elevated oxidative biomarkers and reduced antioxidant capacity support a role for oxidative stress as a mechanistic driver of both cardiovascular and ND progression.

## Nutrition as a modifiable risk factor for noncommunicable diseases with therapeutic potential

Diet, and more particularly dietary patterns rich in health-promoting foods, are increasingly recognised as key modifiable determinants of both cardiovascular and ND trajectories (71, 72). Large cohort studies consistently show that dietary patterns emphasizing fruits and vegetables, whole grains, nuts, fish, and unsaturated fats have been associated with reduced incidence of major cardiovascular events, better cardiometabolic profiles, and lower risk of cognitive decline and dementia (73–75). Within this context, functional or nootropic foods should not be viewed as isolated “magic bullets,” but rather as foods and food matrices that deliver bioactive components capable of targeting shared mechanisms, including dyslipidaemia, endothelial dysfunction, oxidative stress, and chronic low-grade inflammation. By acting on these convergent pathways, brain health-promoting functional foods have the potential to simultaneously modify vascular risk and neurodegenerative processes across the lifespan (76–78).

Positioning such foods within evidence-based dietary patterns, rather than as standalone commercial products, offers a realistic, mechanistically grounded avenue to influence the onset, progression, and possibly treatment response or disease trajectory of both cardiovascular and ND. However, some heritable ND diseases are unlikely to respond substantially to nutritional intervention alone, although nutrition may still provide supportive benefits. Representative dietary models including the Mediterranean (79, 80), DASH (81, 82), and MIND (83, 84) diets consistently demonstrate benefits for cardiometabolic health and are increasingly associated with reduced risk of cognitive decline and dementia.

## Anti-inflammatory roles of nutrients in cardiovascular diseases

A substantial body of evidence supports the role of nutrients and bioactive compounds in modulating inflammatory pathways central to CVD. Many cardioprotective nutrients exert pleiotropic effects, including suppression of pro-inflammatory signalling, improvement of endothelial function, inhibition of platelet aggregation and promotion of inflammatory resolution (85, 86). Several nutrient classes have been investigated for their anti-inflammatory effects preclinically and clinically. Here, we discuss nutrients with shared relevance to CVD and ND.

Long-chain omega-3 polyunsaturated fatty acids (PUFAs), particularly eicosapentaenoic acid (EPA) and docosahexaenoic acid (DHA), are among the most extensively studied. Incorporated into cell membranes, they influence membrane dynamics and signalling pathways, and act as precursors to specialised pro-resolving mediators (SPMs), including resolvins and protectins, which are thought to actively initiate resolution of inflammation (87). These effects are associated with reduced inflammatory markers, inhibition of NF-κB activation, and improved vascular function (88). EPA and DHA in phospholipid forms may exert more potent anti-inflammatory effects due to greater bioavailability (89) and these types of lipids have been implicated in the prevention of the pro-inflammatory effects of bioactive lipids such as platelet-activating factor (PAF) through antagonistic effects in both CVD (90) and ND (18). Generally in the field, dose and formulation inconsistencies remain unresolved regarding which formulations may exert the greatest benefits (91–93). Furthermore, trial findings on the cognitive benefits of omega-3 fatty acids remain mixed (94, 95).

Another promising dietary component in the prevention of CVD and ND is fibre, particularly fermentable forms, which exert anti-inflammatory effects via the gut microbiota. Microbial fermentation produces short-chain fatty acids, which regulate immune responses and enhance gut barrier integrity, limiting endotoxin-driven inflammation (96, 97). In particular, cereal dietary fibre intake was associated with lower levels of inflammatory markers and lower risk of CVD in over 4,000 older U.S. adults (98). However, some evidence suggests that the cardiometabolic benefits associated with short-chain fatty acids may not translate into equivalent benefits for brain health (99).

Conversely, one potential group of dietary bioactive nutrients that may also play a role in such associations are polyphenols. Polyphenols, such as flavonoids, found in plant-based foods, modulate inflammatory signalling through inhibition of NF-κB and activation of Nrf2, reducing oxidative stress and improving endothelial function (100). Decades of research have associated polyphenols with potential vascular benefits. However, polyphenols are often found in forms that have poor bioavailability, but this can be improved through methods such as encapsulation (101). Other nutrients, including vitamin D, zinc, and magnesium, also contribute to immune regulation and are associated with lower inflammatory burden, although evidence for clinical outcomes remains variable (102, 103). Zinc may also play a contributory role through its anti-inflammatory and neuroprotective functions. As an essential trace element involved in redox regulation and immune signalling, zinc modulates inflammatory pathways including NF-κB activation and cytokine release (104). Perturbations in zinc homeostasis have been implicated in Alzheimer’s disease, where abnormal zinc levels may promote amyloid-*β* aggregation and exacerbate neuroinflammation (105). Vitamin D deficiency has been linked to several ND and to immune dysregulation associated with multiple sclerosis (MS) (106). Low serum vitamin D levels may contribute to the pathogenesis of Alzheimer’s disease by worsening amyloid-*β* associated neurodegeneration in older adults (107). Moreover, calcitriol, the active form of vitamin D, demonstrates anti-inflammatory and neuroprotective properties in Parkinson’s disease, potentially mediated through its ability to enhance the expansion of regulatory T cells in mice (108, 109). Another class of nutrients with established links to both CVD and ND are B vitamins, particularly folate, vitamin B6, and vitamin B12, which are central to one-carbon metabolism and regulation of homocysteine. Elevated homocysteine is recognised as a modifiable risk factor associated with vascular dysfunction through mechanisms involving oxidative stress, endothelial damage, and inflammatory signalling. In the central nervous system, increased homocysteine has been implicated in neurotoxicity and is associated with processes relevant to neurodegeneration, including promotion of amyloid-β accumulation and tau-related pathology. Importantly, the evidence supports a causal role for homocysteine in disease progression, with B vitamin supplementation shown to lower homocysteine levels and potentially contribute to disease prevention, particularly when implemented at earlier stages (110). These findings suggest that maintaining adequate B vitamin status may be important for modulating inflammation-mediated vascular and neurodegenerative pathways.

Together, these nutrients act within dietary patterns rich in unsaturated fats, fibre, and plant bioactives. Their combined effects on inflammatory pathways represent a key mechanism linking diet to cardiovascular risk, with potential relevance for neurovascular and neurodegenerative health.

### Functional foods and product development for cardiovascular diseases

Over the past decades, functional foods targeting cardiovascular risk have been developed around several well-characterized mechanisms, including cholesterol reduction, blood pressure control, antithrombotic effects, and improvement of endothelial function (111–113). Examples include foods fortified with long-chain omega-3 fatty acids, plant sterols and stanols, fibre, and specific bioactive peptides, often delivered through spreads, dairy and dairy analogue products, cereal products, and beverages (112, 114). These products have typically been evaluated against conventional cardiovascular endpoints, such as LDL cholesterol, triglycerides, blood pressure, and markers of platelet activation, with many demonstrating modest but clinically meaningful improvements in risk factors when incorporated into habitual diets. However, most cardiovascular-oriented functional foods have been conceptualized with a single organ focus, rarely considering neurocognitive outcomes or neurovascular health (111, 115). Given the intimate links between cardiometabolic status, vascular integrity, and brain function, there is an opportunity to redesign and reevaluate cardiovascular functional foods using dual cardiovascular and neurocognitive endpoints (115, 116). Future product development should explicitly consider how lipid profiles, anti-inflammatory effects, and endothelial benefits induced by these foods might translate into preservation of cognitive function and reduced risk of ND, thereby bridging traditional “heart healthy” claims with emerging brain health–promoting potential given the strong cardio-neuro associations discussed here.

## Nutrient roles in neurodegenerative diseases

In contrast to the relatively mature field of nutrition and cardiovascular disease, nutritional strategies for ND such as Alzheimer’s disease, Parkinson’s disease, and vascular dementia remain fragmented and comparatively underdeveloped (117–119). Much of the current evidence for diet and neurodegeneration arises from observational studies, which, although informative, are vulnerable to confounding, reverse causation, and difficulties capturing long-term dietary patterns (119). Interventional studies have often focused on single nutrients or high-dose supplements, such as isolated vitamins, antioxidants, or omega-3 capsules, with generally modest and sometimes inconsistent effects on cognitive outcomes (120, 121). Moreover, trials frequently enrol heterogeneous populations with respect to disease stage, vascular comorbidities, and genetic background, and they rarely integrate comprehensive vascular endpoints or neuroimaging markers to clarify mechanisms (122, 123). There is also limited attention to real-world issues such as food format, palatability, and cultural acceptability, which are critical for adherence among specific populations, such as the aging population or cognitively impaired individuals (124, 125). Collectively, these gaps highlight the need to move beyond single-nutrient, supplement-driven approaches towards rigorously designed, food- and pattern-based interventions that explicitly target shared vascular and neurodegenerative pathways over sufficiently long timeframes.

### Functional foods targeting neurodegenerative diseases (ND)

Despite current limitations in nutritional strategies for ND, several dietary patterns and food categories show promise as brain health-promoting functional or nootropic foods. Patterns such as the Mediterranean and MIND diets, characterized by high intakes of fruits and vegetables, whole grains, legumes, nuts, fish, and extra-virgin olive oil, alongside reduced consumption of saturated fats and ultra-processed foods, have been consistently associated with slower cognitive decline and reduced risk of Alzheimer’s disease and dementia (80, 84). These dietary patterns provide a complex matrix of unsaturated lipids, polyphenols, vitamins, and minerals that influence neuronal membrane composition, synaptic plasticity, neuroinflammatory signalling, and vascular function. Within these patterns, specific food groups appear particularly relevant. Marine foods and other sources of long-chain omega-3 fatty acids support neuronal integrity and contribute to the formation of the controversial (126) pro-resolving lipid mediators involved in vascular and neuroinflammatory regulation (127). Standalone, long-chain omega-3 fatty acids and phospholipid preparations are under investigation to tackle NDs (91, 117, 128). Polyphenol-rich foods, including berries, cocoa, olives, and herbs, exert antioxidant and anti-inflammatory effects while modulating endothelial function, mitochondrial health, and cellular aging (129). While the potential antioxidant effects of polyphenol-rich sources are often touted as potential strategies to target ND, human studies have largely led to mixed or negative conclusions (130, 131), potentially because of their known poor bioavailability (101). Fermented and fibre-rich foods are also thought to contribute nutrients with neuroprotective effects by influencing the gut–brain axis and systemic inflammation, although mechanistic and clinical evidence in ND contexts remains limited (132). In the context of brain health, it is worth noting that some of these functional foods and their bioactive components are often described as “nootropic” in popular discussions; however, their effects extend beyond cognition, learning, and memory. Rather, they act through pathways central to both cardiovascular and neurodegenerative health, including lipid metabolism, endothelial function, and chronic inflammation (22). Accordingly, “nootropic foods” are better conceptualized within broader dietary strategies that integrate cardiometabolic and neuroprotective benefits, highlighting the need for evaluation using combined vascular and neurocognitive endpoints.

## Future directions and limitations

**Figure 2:**
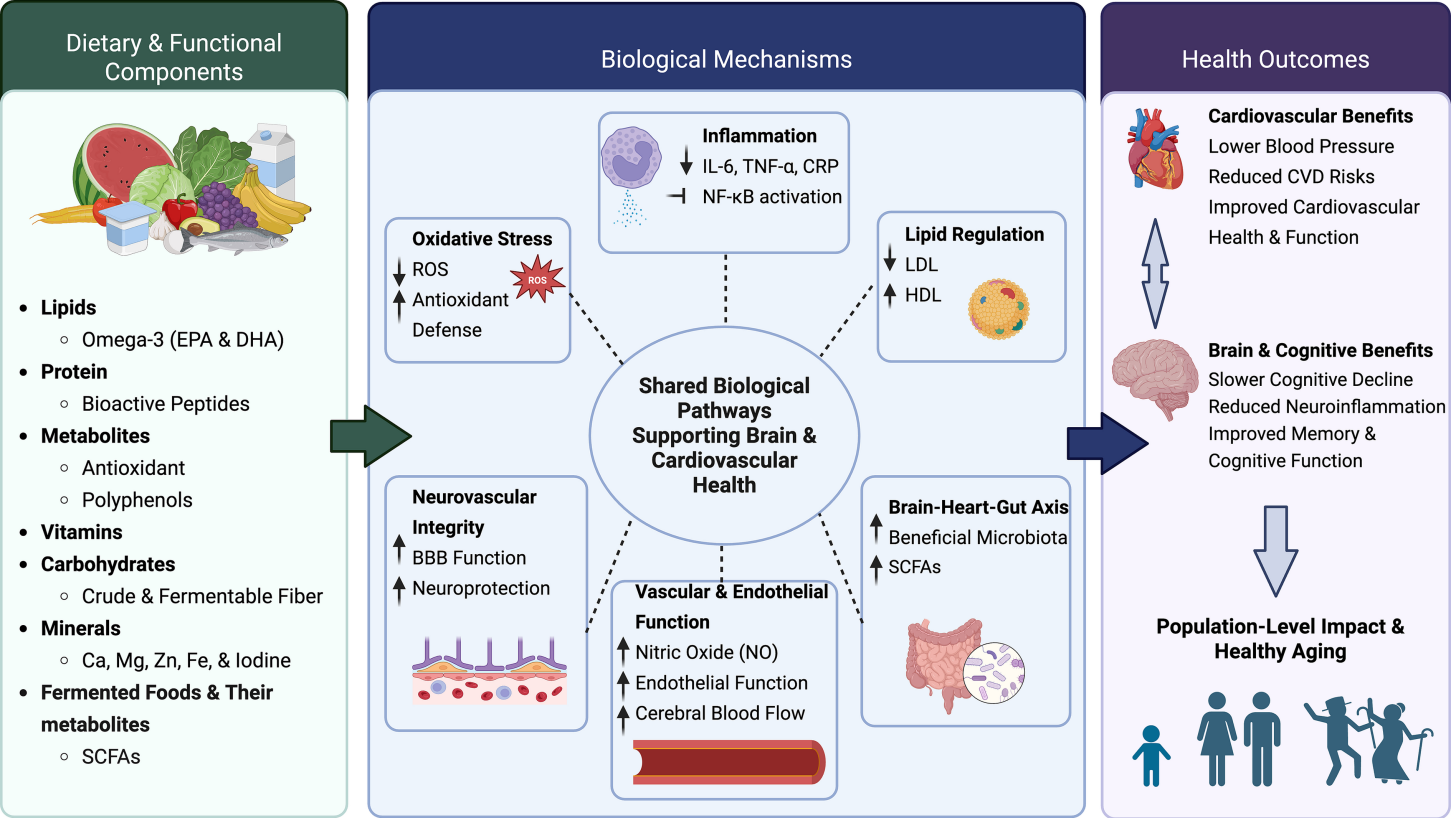
Dietary interventions and bioactive nutrients target shared pathways to improve cardiovascular and neurodegenerative health. Abbreviations: BBB, brain–blood barrier; CRP, C-reactive protein; CVD, cardiovascular disease; DHA, docosahexaenoic acid; EPA, eicosapentaenoic acid; HDL, high-density lipoprotein; IL-6, interleukin-6; LDL, low-density lipoprotein; NF-κB, nuclear factor kappa B; NO, nitric oxide; ROS, reactive oxygen species; SCFAs, short-chain fatty acids; TNF-*α*, tumour necrosis factor-alpha.

Despite common mechanisms in NCD, there is no magic bullet to tackle these complex conditions. However, it is clear that there are nutritional strategies that may reduce the risk of conditions associated with systemic inflammation, including NCD like ND and CVD. These strategies are highlighted in Figure 2.

### Personalized nutritional strategies for non-communicable diseases

As evidence accumulates for the role of diet in modulating both cardiovascular and neurodegenerative risk, the next step is to move from ‘one size fits all’ recommendations toward more personalized nutritional strategies, particularly when implemented earlier in life before disease onset. Advances in nutrigenetics, metabolomics, lipidomics, and microbiome research offer the potential to identify subgroups that are particularly responsive or resistant to specific dietary patterns or functional food interventions (133, 134). For example, genetic variants related to lipid transport, inflammation, or amyloid processing, as well as baseline cardiometabolic profiles, may modulate individual responses to diets rich in unsaturated fats, marine foods, or polyphenol-dense plant foods (135, 136). Integrating these biological signatures with granular dietary data and digital health tools may allow tailoring of brain health-promoting functional foods to an individual’s vascular risk, cognitive status, and lifestyle context within an integrated cardio-neuro framework (137). Future trials should, therefore, incorporate stratified designs to evaluate how personalized combinations of foods and dietary patterns influence both cardiovascular markers and cognitive or neurobiological outcomes. Such an approach could help to optimize efficacy, minimize unnecessary supplementation, and move functional and nootropic foods from generic “heart healthy” or “brain healthy” labels toward truly targeted interventions along the cardio–neuro continuum, and strategically aimed at potentially reducing systemic inflammation.

One converging theme across NCDs is the emerging concept of a gut-brain-heart axis in which gut microbiota-derived metabolites act as systemic mediators linking vascular, inflammatory, and neurodegenerative pathology (138, 139). Dysbiosis and increased gut permeability facilitate the escape of microbially derived metabolites such as SCFAs and trimethylamine N-oxide (TMAO) into the circulation, where they modulate endothelial function, neurohumoral signalling, and neuroinflammation (140, 141). SCFAs (i.e., acetate, propionate, and butyrate) are generally regarded as beneficial metabolites that support barrier integrity, promote regulatory immune responses, and dampen pro-inflammatory cytokine production (142, 143). In contrast, elevated TMAO, arising from microbial metabolism of dietary choline and carnitine to trimethylamine (TMA), has been reproducibly associated with incident cardiovascular events, vascular dysfunction, and mortality, and is increasingly linked to cognitive decline and vascular cognitive impairment through effects on endothelial injury, cholesterol handling, and neuroinflammatory pathways (144–147). Against this background, diet emerges as a key modifiable lever: high-fiber, plant-rich and fermented-food dietary patterns promote SCFA-producing taxa and a more favorable metabolite profile, whereas Western diets enriched in red and processed meat increase TMA precursors, foster dysbiosis, and raise TMAO levels, suggesting that microbiome-directed nutritional strategies may help to attenuate gut-brain-heart axis-mediated cardiovascular and neurodegenerative risk (144, 148). Furthermore, as demonstrated in the patent landscape, there is increased interest in developing probiotics and similar products that target gut dysbiosis and inflammatory diseases that may benefit the cardio-neuro axis (149).

### Current limitations and potential for new functional foods and nutritional strategies

Taken together, the current evidence base supports an increasingly coherent role for brain health-promoting functional foods in shaping the risk and progression of both cardiovascular and ND, but several limitations must be acknowledged. Many studies are observational, rely on self-reported dietary intake, and are conducted within specific cultural or socioeconomic contexts that may limit generalizability (150, 151). Interventional trials are often short in duration relative to the long prodromal phase of neurodegenerative disorders, focus on single nutrients rather than whole food matrices, and frequently lack integrated assessment of vascular and neurocognitive outcomes (120, 122, 152). Regulatory frameworks and commercial pressures often promote narrow, claim-focused product development rather than fostering mechanistically grounded, clinically meaningful innovation (153, 154). Despite these challenges, the convergence of mechanistic, epidemiological, and early interventional data suggests substantial potential for functional or nootropic foods, beverages, and/or dietary patterns rich in unsaturated lipids, fibre, and bioactive plant compounds to modulate shared inflammatory and lipid-related pathways across cardiovascular and neurodegenerative conditions. Realizing this potential will require well-designed, long-term studies, thoughtful product development, and a shift from marketing-oriented concepts toward rigorous, validated health-promoting dietary strategies embedded in everyday food environments and traditions.

Furthermore, future progress in this area will require interdisciplinary collaboration among cardiologists, neurologists, nutrition scientists, systems biologists, microbiome researchers, and public health experts. Integrated research frameworks combining dietary assessment, vascular biomarkers, neuroimaging, multiomics approaches, and clinical outcomes will be essential for validating the proposed cardio-neuro axis and translating mechanistic insights into effective prevention and intervention strategies. Such collaborative efforts may help accelerate the development of personalized nutritional approaches that simultaneously target cardiovascular and neurodegenerative health across the life course.

In conclusion, cardiovascular and neurodegenerative diseases have traditionally been considered distinct clinical entities, yet accumulating evidence indicates that they share interconnected inflammatory, vascular, metabolic, and lipid-mediated mechanisms. We propose that these interactions can be conceptualized as a cardio-neuro axis, providing a framework for understanding how dietary exposures influence both cardiovascular and brain health across the life course. Although nutritional strategies for cardiovascular disease are comparatively mature, translation to neurodegenerative disease remains fragmented and frequently focused on isolated nutrients rather than integrated dietary patterns. Future studies should adopt whole-diet approaches, incorporate vascular and neurocognitive endpoints, and leverage emerging personalized nutrition and multiomics tools. Embedding nutrition within a unified cardio-neuro framework may offer a scalable and mechanistically grounded strategy to reduce the growing burden of NCDs.

## Data Availability

The original contributions presented in the study are included in the article/supplementary material, further inquiries can be directed to the corresponding author/s.
